# How can plant genetic engineering contribute to cost-effective fish vaccine development for promoting sustainable aquaculture?

**DOI:** 10.1007/s11103-013-0081-9

**Published:** 2013-06-01

**Authors:** Jihong Liu Clarke, Mohammad Tahir Waheed, Andreas G. Lössl, Inger Martinussen, Henry Daniell

**Affiliations:** 1 Bioforsk, Norwegian Institute for Agricultural and Environmental Research, Ås, Norway; 2 Department of Biochemistry, Faculty of Biological Sciences, Quaid-i-Azam University, Islamabad, 45320 Pakistan; 3 Department of Crop Sciences, University of Natural Resources and Applied Life Sciences, Vienna, Austria; 4 Departments of Biochemistry and Pathology, University of Pennsylvania School of Dental Medicine, 240 South 40th St, 547 Levy Building, Philadelphia, PA 19104 USA

**Keywords:** Aquaculture, Food security, Fish vaccine, Plant genetic engineering, Regulatory constraints

## Abstract

Aquaculture, the fastest growing food-producing sector, now accounts for nearly 50 % of the world’s food fish (FAO in The state of world fisheries and aquaculture. FAO, Rome, 2010). The global aquaculture production of food fish reached 62.7 million tonnes in 2011 and is continuously increasing with an estimated production of food fish of 66.5 million tonnes in 2012 (a 9.4 % increase in 1 year, FAO, www.fao.org/fishery/topic/16140). Aquaculture is not only important for sustainable protein-based food fish production but also for the aquaculture industry and economy worldwide. Disease prevention is the key issue to maintain a sustainable development of aquaculture. Widespread use of antibiotics in aquaculture has led to the development of antibiotic-resistant bacteria and the accumulation of antibiotics in the environment, resulting in water and soil pollution. Thus, vaccination is the most effective and environmentally-friendly approach to combat diseases in aquaculture to manage fish health. Furthermore, when compared to >760 vaccines against human diseases, there are only about 30 fish vaccines commercially available, suggesting the urgent need for development and cost-effective production of fish vaccines for managing fish health, especially in the fast growing fish farming in Asia where profit is minimal and therefore given high priority. Plant genetic engineering has made significant contributions to production of biotech crops for food, feed, valuable recombinant proteins etc. in the past three decades. The use of plants for vaccine production offers several advantages such as low cost, safety and easy scaling up. To date a large number of plant-derived vaccines, antibodies and therapeutic proteins have been produced for human health, of which a few have been made commercially available. However, the development of animal vaccines in plants, especially fish vaccines by genetic engineering, has not yet been addressed. Therefore, there is a need to exploit plant biotechnology for cost effective fish vaccine development in plants, in particular, edible crops for oral fish vaccines. This review provides insight into (1) the current status of fish vaccine and vaccination in aquaculture, (2) plant biotechnology and edible crops for fish vaccines for oral administration, (3) regulatory constraints and (4) conclusions and future perspectives.

## Introduction

Fish is an excellent animal protein source and contains a wide range of essential human nutrients. Up to 80 % of the world’s fish production is used for human consumption, indicating the important role of aquaculture for food security. Fisheries and aquaculture play also an important role in the livelihoods of millions of people worldwide from the small-scale inland fishermen who harvest fishes from lakes and rivers to the industrial scale fish farming. Thus, sustainable fish farming contributes considerably to food security (www.fao.org).

**Fig. 1:**
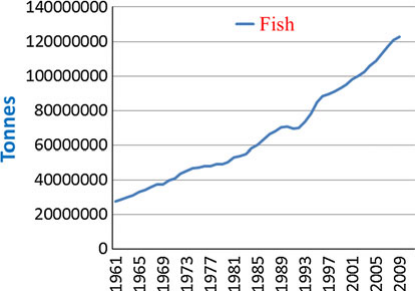
Worldwide fish production in five decades. Data source (www.fao.org/fishery/aquaculture)

Aquaculture is the farming of aquatic organisms including fish, crustaceans, molluscs and aquatic plants. Fisheries and aquaculture make important contributions to the human population as protein sources. The global aquaculture production of food fish reached 62.7 million tonnes in 2011 and is continuously increasing with an estimated production of food fish of 66.5 million tonnes in 2012 (a 9.4 % increase in 1 year, FAO, www.fao.org/fishery/topic/16140). In the past five decades, the world fish supply has rapidly increased with an average growth rate of 3.2 % per year and constitutes an important source of nutrition and animal protein for humans (FAO 2012; http://www.fao.org, Fig. 1). This is particularly the case in Asia, where approximately 90 % of the total global aquaculture products comes from. Among the Asian countries, China alone produces ca. 70 % of the world total volume of aquaculture products and has become the largest producer of farmed seafood in the world, with an increase of 490 % since 1978 (Ellis 2009). It is estimated that in the next decade total production from both capture and aquaculture will exceed that of beef, pork or poultry. Due to higher demand for fish, world fisheries and aquaculture production are projected to reach about 172 million tonnes in 2021, of which aquaculture is projected to reach about 79 million tonnes, rising by 33 % over the period 2012–2021 (FAO 2012, http://www.fao.org). This boom in aquaculture will help to achieve certain millennium development goals either directly (e.g. eradication of extreme poverty and hunger) or indirectly (e.g. substantial improvement in economies). However, aquaculture is as vulnerable to adverse impacts of disease and unfavourable environmental conditions as is farming of other animals. Disease outbreaks in recent years have affected Atlantic salmon, oyster and marine shrimp farming in several countries of the world, resulting in partial or sometimes total loss of production. In 2010, aquaculture in China suffered production losses of 1.7 million tonnes caused by natural disasters, diseases and pollution. Disease outbreaks virtually wiped out marine shrimp farming production in Mozambique in 2011 (FAO 2012). Fish diseases not only pose a threat to the aquaculture industry but also to human livelihood and health. Apart from zoonoses, use of certain chemicals and antibiotics for fish health also pose certain risks to the environment, human health and food security (for a review see Sapkota et al. 2008). Management of aquatic animal health is therefore an important issue for food security, to protect livelihoods of millions of people, the aquaculture industry and the environment.

## History and current status of fish vaccines and vaccination approaches

Compared with human vaccine history starting from the discovery of vaccination by Edward Jenner in 1796 leading to production of >760 vaccines for protecting human health, fish vaccine development has a very short history with roughly 40 years since the 1970s. It took over three decades from the first scientific report describing fish vaccination using an inactivated orally administrated *Aeromonas salmonicida* vaccine. The first licensed fish vaccine was made commercially available in 1976 (Evelyn 1997). The first fish vaccine was produced from killed *Yersinia ruckeri*, and used to protect fishes against enteric redmouth (ERM) by oral administration. Soon after the release of ERM oral fish vaccine, a new vaccine for *Vibrio anguillarum* became available in the USA with vaccination via immersion. These were followed with the release of Furunculosis vaccines in Europe in the 1980s. To date, there are no more than 30 commercial vaccines for the prevention of a wide range of fish diseases, with a few more under development (Smith 2008). The economic, environmental and animal welfare benefits have been recognised as a result of the widespread use of vaccines in the aquaculture industry. The potential from vaccines for lower mortality, improved growth efficiency and higher yields is now a critical factor in disease management programmes in aquaculture. To date, commercially used fish vaccines are mainly: killed vaccines such as inactivated virus or bacterial antigens, subunit vaccines and recombinant vaccines with humoral (antibody) responses, or live, attenuated and DNA vaccines for cytotoxic T cell response (Plant and LaPatra 2011, http://www.pharmaq.no).

At present, three main delivery approaches are used for fish vaccination: (1) injection vaccination, (2) oral vaccination, and (3) immersion vaccination (including bath and spray vaccination). The advantages and drawbacks of each vaccination method are summarized in Table 1.

**Table 1:** Fish vaccination methods and their advantages and drawbacks [adapted from Plant and LaPatra (2011) and http://www.pharmaq.no]

Vaccination method	Advantages	Drawbacks
Injection Intraperitoneal (adjuvanted or not) Intramuscular (DNA)	Most common method of vaccine delivery in fish Effective in generating both humoral (antibody) and cellular cytotoxic response Protection is of long duration	Not feasible for fishes under 20 g Stressful for the fish due to handling and anesthetizing Labour intensive Expensive (high labour costs or expensive injection machine)
Oral Micro-encapsulation Bio-encapsulation	Ideal delivery method via feed Easiest, no technical skill required No handling stress for the fish Protection from the digestive system No additional labour cost No investment on instrument required Feasible for mass vaccination of all sizes of fishes	Large amount of antigen needed Poor and short-term protection (except for *Y. ruckeri* and *V. anguillarum*). Gastric degradation can affect protective antigen
Immersion by Bath Spray Dip	Simple and suitable for mass vaccination Less stress for the fish than injection Lower labour costs Less risk to vaccination team	Not suitable for all farmed fishes Stressful for the fish because of netting and transportation prior to spray vaccination Large amount of vaccine required in the case of the bath method Lower level of protection and duration of immunity

Given that none of the vaccination approaches are universal, choice of vaccination method will be largely determined by the type and size of the fish, protection required, pathogen’s nature, type of immune reaction required and the vaccine’s nature (single or multivalent), as well as the cost which is essential for fish industry and small fish farmers in the developing countries. New fish vaccine development and the production of fish vaccines require that vaccines must be effective and suitable for large-scale production at low cost, making the vaccines available and affordable to the aquaculture fish industry and small fish farmers in the developing countries.

Facing economic pressure in the aquaculture industry, intensive fish farming has increased the spread of diseases and parasites. To manage the problem, a large amount of antibiotics are applied in aquaculture with the hope of enhancing production and improving the socioeconomic profile of the fish farmers in the developing countries.

The presence of antibiotic compounds in the aquatic environment has resulted in environmental pollution, especially water pollution. It leads also to the development of antibiotic-resistant bacteria making bacterial disease control in aquaculture a challenging task. Increased mortality of *Penaeus monodon* larvae due to antibiotic-resistant *Vibrio harveyi* infection has been reported (Karunasagar et al. 1994). Furthermore, infectious diseases in aquaculture are caused not only by bacteria but also viruses, fungi and parasites. Thus, vaccination is the most effective way to protect fishes and to manage aquaculture in an environmentally friendly manner. Application of plant biotechnological tools for fish vaccine development is of importance for aquaculture as the fish vaccines have to be produced at a low cost and for easy scaling up, making them accessible and affordable for not only the aquaculture industry worldwide, but in particular for improvement of the conditions for small fish farmers in the developing countries.

## Exploitation of plant genetic engineering for low cost production of fish vaccines

Fish have a functional immune system similar to mammals (innate and adaptive) and the advancement and experiences of plant biotechnology in vaccine development for humans and other mammals could be of importance for the development of fish vaccines. The use of plants for development and production of recombinant vaccines offers several advantages. Plant-based systems are more economical as plants can be grown on a larger scale than in other systems. Low cost is no doubt one of the most important issues in the future development of fish vaccines. Plants also possess the ability to carry out post-translational modifications similar to naturally occurring systems. The plant-based systems bypass the safety concerns inherent in live virus vaccines.

To date, three main plant-based techniques have been used for the expression of a large number of vaccine antigens, monoclonal antibodies (mAbs) and other biopharmaceuticals in plants. These are (1) stable expression of transgenes in the nuclear genome of transgenic plants or cell culture, (2) stable expression of transgenes in the plastid genome of transplastomic plants by plastid genetic engineering and (3) transient expression of transgenes in plants. A number of reviews have covered all the three methodologies (Streatfield 2007; Daniell et al. 2009; Clarke and Daniell 2011; Lössl and Waheed 2011; Maliga and Bock 2011; Yusibov et al. 2011). Each system has its advantages and limitations and the method of choice is largely depending on what kind of fish vaccines are to be expressed, as briefly described in Table 2. To date, both food and non-food crops (especially tobacco plant) have been used for the development of a number of animal vaccines, such as a poultry vaccine against Newcastle disease (Hahn et al. 2007; Yang et al. 2007; Li et al. 2007; Gómez et al.^2009^; Van Eck and Keen 2009; Wu et al. 2009: for reviews see Floss et al. 2007 and He et al. 2008), rabies (Ashraf et al. 2005; Loza-Rubio et al. 2008; Roy et al. 2010; Loza-Rubio et al. 2012), Porcine reproductive and respiratory syndrome virus (PRRSV) and Porcine post-weaning diarrhea in piglets (Chen and Liu 2011; Kolotilin et al. 2012). The vaccine against Newcastle disease was the first plant-made animal vaccine receiving regulatory approval from the US Department of Agriculture (USDA) Center for Veterinary Biologics in 2006 (www.thepoultrysite.com/poultrynews/8949/usda-issues-license-for-plant-cell-producednewcastle-disease-vaccine-for-chickens; Joensuu et al. 2008).

**Table 2:** Plant expression systems and their future application in fish vaccine development

Plant expression system	Fish vaccine for oral vaccination	Fish vaccine for injection	Fish vaccine for immersion
Transient expression Advantages: Fast and easy scaling up Feasible for *Nicotiana benthamiana* and tobacco plants Limitations: Not applicable in edible crops	Not feasible	Feasible and desirable	Feasible and desirable
Plastid engineering Advantages High level expression of foreign proteins (>70 % of total soluble proteins), suitable for production in large quantity Biosafety via maternal inheritance and inducible promoter like T7 Methods established in both food (lettuce, soybean, tomato, potato, cabbage etc.) and non-food crops (tobacco, poplar etc.) Multiple genes (up to 8 genes) can be expressed in a single event Cost effective Limitations Not applicable for glycoproteins Protein stability at room temperature	Feasible and desirable for both single and multivalent vaccines	Feasible and suitable for both single and multivalent vaccines	Feasible and desirable for both single and multivalent vaccines An example: fish vaccine antigen expressed in tobacco chloroplasts (Clarke et al. unpublished results)
Nuclear genetic engineering Advantages Methods established in a large number of food and non-food crops Easy and feasible Limitations Low expression level of recombinant proteins Biosafety concern as pollen contains transgene Transgene segregation when via seed propagation	Feasible but less desirable due to the low expression level Report: Antigen fused with LTB and expressed in potato showed humoral immune response in carp gut (Companjen et al. 2005)	Feasible and suitable	Feasible but less desirable due to the low expression level

Moreover, studies on plant-based animal vaccines for protecting mink, dogs, and cats are reported (Dalsgaard et al. 1997; Molina et al. 2004). Molina et al. (2004, 2005) demonstrated high-level expression of a tobacco chloroplast-derived vaccine based on a B cell epitope from canine parvovirus and the induction of neutralizing antibodies. Three recent reviews by Floss et al. (2007), Joensuu et al. (2008) and Rybicki (2010) have provided an overview of production of veterinary vaccines in plants. However, plants as expression systems for production of fish vaccines are lagging behind compared with the plant-made veterinary vaccines for non-aquatic (land-based) animals. Based on the special advantages of oral vaccination in aquaculture, Companjen et al. (2005) successfully expressed the non-toxic part of the *E. coli* heat-labile enterotoxin LTB fused with a viral peptide or GFP in potato tuber for oral immunization and induction of specific humoral immune response in carp upon feed-mediated administration. This study demonstrated the feasibility of producing fish vaccines for oral vaccination in an edible crop and the technology shall be explored further. To boost an efficient delivery of plant-made oral fish vaccine to immune-competent cells in the gut mucosa, a carrier molecule i.e. LTB in the study was fused to the oral vaccine antigens to stimulate the uptake and immune response upon feed-mediated oral immunization.. Another attempt is our own ongoing research in production of a fish vaccine in tobacco chloroplasts against viral nervous necrosis (VNN) caused by Nodavirus (Clarke et al. ongoing research). VNN affects farmed fish such as turbot, Atlantic halibut and Atlantic cod, as well as wild fish (Grotmol et al. 1995, 1997; Munday et al. 2002; Sommerset et al. 2005). The risk of VNN spreading from escapes of farmed fish to wild indicates the significance of the development of a cost effective and safe vaccine against VNN infection. The economic importance of such a vaccine for farmed fish is self-evident. In this study, transplastomic tobacco lines expressing RNA2 as the antigen candidate were produced and are currently subject to various molecular analyses (Clarke et al. unpublished results).

## Engineering edible crops for the development of fish vaccines for oral immunization

Edible crops are ideal green factories for the production of therapeutic proteins and vaccines for oral immunization. In aquaculture, among the current fish vaccination methods shown in Table 1, a fish vaccine produced in an edible crop (or microalgae) for oral immunization is undoubtedly advantageous because oral vaccination of fish is an easy, labour-saving and stress free method which is suitable for all fishes independent of the fish size.

Despite the advantages and potentials of plant vaccine production systems for animal health including aquatic animals, there are only a few studies reporting the veterinary vaccine antigens expressed in edible crops, only one case for fish vaccine produced in potato for oral delivery via feed suggesting strongly that research effort is needed to develop and advance the research field in the future for effective management of fish health by cost effective plant-made oral fish vaccines. Successful management of fish health will directly contribute to sustainable food fish production in the future. To date, lettuce and potato tubers have been used for the development of plant-based animal vaccines (Companjen et al. 2005; Gómez et al. 2008; Matsui et al. 2009). So far, there is no report describing fish vaccine antigens expressed in edible crops by plastid genome engineering, despite the technology for plastid engineering of edible crops such as lettuce, tomato, potato, cabbage etc. having been developed and used to express a number of foreign proteins (Kanamoto et al. 2006; Ruf et al. 2001; Ruhlman et al. 2007, 2010; Daniell et al. 2009; Cardi et al. 2010; Clarke and Daniell 2011; Davoodi-Semiromi et al. 2010; Kanagaraj et al. 2011; Boyhan and Daniell 2010; Lakshmi et al. 2013). Based on the experience from human vaccines produced in edible crops, the development of fish vaccines in edible crops for oral vaccination will be a reality in the future.

## Regulatory constraints

It was 30 years ago when the first genetically modified (GM) plant was produced by using *Agrobacterium tumefaciens*—mediated genetic transformation (for historical perspective see Bevan et al. 1983; Fraley et al. 1983; Herrera-Estrella et al. 1983 and review by Vasil 2008). Thirteen years later, the first GM crop was commercialized in 1996 (http://www.isaaa.org). Since then, there has been a fast development with first, second and third generations of GM plants produced worldwide. Despite the encouraging news that the global status of commercialized biotech crops has reached 170.3 million hectares globally in 2012 (http://www.isaaa.org), at an annual growth rate of 6 %, up 10.3 million from 160 million hectares in 2011 and with significant benefits for farmers, the regulatory constraints are a well-known hurdle for commercialization of biotech crops in many countries, especially in Europe. Molecular farming using plants or plant cell lines as a green factory to produce vaccines and biopharmaceuticals has also made considerable progress with commercially released plant-made therapeutic proteins, and a number of vaccines and therapeutic proteins are undergoing clinical trials or are in the pipeline to be approved (Yusibov et al. 2011; http://www.genengnews.com/gen-news-highlights/); however, it has encountered the same regulatory constraints as other GM crops. Under the current regulatory requirements, it’s estimated that it takes on average 7-10 million euros to approve a GM crop for cultivation (Paul et al. 2011).

Current USDA-APHIS regulatory requirements are based on the use of plant pathogens (*Agrobacterium*) for transformation or use of plant pathogenic sequences (*Agrobacterium* genome sequence or plant viral genome sequence, especially the CaMV promoter). So, in order to minimize regulatory costs, one could use the chloroplast transformation approach for molecular pharming, which doesn’t use any plant pathogenic sequences. This approach should significantly minimize the cost of regulatory approval for field studies. Indeed, plant-made pharmaceuticals engineered via the chloroplast genome have been tested in the field several years ago (Arlen et al. 2007). One among the most important regulatory hurdles for molecular pharming is transgene containment. Early plant-made vaccine companies were shut down by USDA-APHIS for contamination of food/feed grains by corn seeds expressing human therapeutic proteins (e.g. Prodigene). Such regulatory challenges could therefore be avoided by not expressing vaccines in seeds. For example, expressing vaccine antigens in leaves facilitates their harvest before appearance of any reproductive structures, thereby avoiding contamination via pollen or seeds (Daniell et al. 2009). In addition, expressing vaccine antigens via the chloroplast genome facilitates maternal inheritance of transgenes and minimizes or eliminates out-cross via pollen (Daniell 2007; Daniell et al. 1998).

Another important cost in regulatory approval is the need for release into the environment, requiring large acreage of field studies in different geographical locations. However, for molecular pharming using the chloroplast transformation approach, high levels of expression result in minimal acreage. For example, one acre of cultivation could produce up to 360 million doses of vaccines (Koya et al. 2005; Watson et al. 2004). Thus, the production could be contained within the greenhouse, eliminating the need for field release.

So far, none of the plant-made vaccines has been approved for oral delivery, an essential requirement for low cost fish vaccine. Bioencapsulation protects vaccine antigens expressed within plant cells, and they are released in the gut by the action of microbes colonizing the gut (Limaye et al. 2006; Kwon et al. 2013a, b; Arlen et al. 2008; Davoodi-Semiromi et al. 2010). However, neither the transient viral expression system that infects plant cells nor low level expression of stable nuclear expression is ideal for oral delivery of vaccines. However, several oral vaccines expressed via the chloroplast genome have been shown to be effective against pathogen or toxin challenge (Davoodi-Semiromi et al. 2010; Arlen et al. 2008) or immune disorders (Ruhlman et al. 2007; Verma et al. 2010). In addition, regulatory agencies require demonstration of long-term stability of vaccine at room temperature. Such stability has been shown by storage of lyophilized leaf materials for several months or years expressing human therapeutic proteins (Kwon et al. 2013a, b), vaccine antigens or autoantigens (Lakshmi et al. 2013, Kwon et al. 2013a, b). Moreover, the process of lyophilization eliminates microbes that colonize plants, an important regulatory requirement (Kwon et al. 2013a). In addition, the concentration of vaccine antigens is increased 15–25 fold, significantly reducing the amount of plant materials required for effective vaccination (Kwon et al. 2013a). The aforementioned advantages make the lettuce chloroplast system ideal for oral vaccines and several human therapeutic proteins have been expressed at high levels in lettuce chloroplasts (Davoodi-Semiromi et al. 2010; Kanagaraj et al., 2011; Boyhan and Daniell 2010; Lakshmi et al. 2013; Ruhlman et al. 2007, 2010). Future studies should therefore focus on edible leaves rather than tobacco that has nicotine and other alkaloids, not permitted by any of the global regulatory agencies.

## Conclusions

With the challenges of the growing world population, food security demand and unpredictable climate change, aquatic fish health and management have become a global concern which affects protein-based food security, the environment, and the aquaculture industry and millions of fish farmers in the developing countries. To use biotechnological tools to manipulate plants for low-cost and safe vaccine production for farmed fish is a research field which needs to be advanced and strengthened. This review has addressed these issues and provided an overview of the current situation in fish health management, the status of fish vaccine and vaccinations, as well as how to explore plant genetic engineering for the development and cost-effective production of fish vaccines. The utilization of plants for low-cost and large quantity production of fish vaccines with oral immunization by plant genetic engineering, especially plastid genetic engineering of edible crops, should be emphasized. Oral vaccination is of special importance for fishes weighing less than 20 g. To promote an efficient delivery of plant-made oral fish vaccine to immune-competent cells in the gut mucosa, a carrier molecule such as LTB or CTB should be fused to the oral vaccine antigens to stimulate the uptake and immune response upon feed-mediated oral immunization. Altogether, there is an urgent need for the research community to advance and implement plant genetic engineering of edible crops for production of fish vaccines for oral vaccination via feed.
